# Feasibility and coverage of implementing intermittent preventive treatment of malaria in pregnant women contacting private or public clinics in Tanzania: experience-based viewpoints of health managers in Mkuranga and Mufindi districts

**DOI:** 10.1186/1472-6963-13-372

**Published:** 2013-10-01

**Authors:** Godfrey M Mubyazi, Pascal Magnussen, Jens Byskov, Paul Bloch

**Affiliations:** 1 Department of Health Systems and Policy Research and Centre for Enhancement of Effective Malaria Interventions (CEEMI), National Institute for Medical Research (NIMR), 2448 Ocean Road, P.O Box 9653, Dar es Salaam, Tanzania; 2 Amani Medical Research Centre, Muheza, P.O Box 81, Tanga, Tanzania; 3 DBL - Centre for Health Research and Development, Institute of Veterinary, Pathobiology, Faculty of Life Sciences, University of Copenhagen, Copenhagen, Denmark; 4 Steno Diabetes Center, Steno Health Promotion Center, Gentofte, Denmark

**Keywords:** Intermittent preventive treatment, Sulphadoxine-pyrimethamine, Malaria, Pregnancy, Antenatal care, Health systems, Health services, Tanzania

## Abstract

**Background:**

Evidence on healthcare managers’ experience on operational feasibility of malaria intermittent preventive treatment for malaria during pregnancy (IPTp) using sulphadoxine-pyrimethamine (SP) in Africa is systematically inadequate. This paper elucidates the perspectives of District Council Health Management Team (CHMT)s regarding the feasibility of IPTp with SP strategy, including its acceptability and ability of district health care systems to cope with the contemporary and potential challenges.

**Methods:**

The study was conducted in Mkuranga and Mufindi districts. Data were collected between November 2005 and December 2007, involving focus group discussion (FGD) with Mufindi CHMT and in-depth interviews were conducted with few CHMT members in Mkuranga where it was difficult to summon all members for FGD.

**Results:**

Participants in both districts acknowledged the IPTp strategy, considering the seriousness of malaria in pregnancy problem; government allocation of funds to support healthcare staff training programmes in focused antenatal care (fANC) issues, procuring essential drugs distributed to districts, staff remuneration, distribution of fANC guidelines, and administrative activities performed by CHMTs. The identified weaknesses include late arrival of funds from central level weakening CHMT’s performance in health supervision, organising outreach clinics, distributing essential supplies, and delivery of IPTp services. Participants anticipated the public losing confidence in SP for IPTp after government announced artemither-lumefantrine (ALu) as the new first-line drug for uncomplicated malaria replacing SP. Role of private healthcare staff in IPTp services was acknowledged cautiously because CHMTs rarely supplied private clinics with SP for free delivery in fear that clients would be required to pay for the SP contrary to government policy. In Mufindi, the District Council showed a strong political support by supplementing ANC clinics with bottled water; in Mkuranga such support was not experienced. A combination of health facility understaffing, water scarcity and staff non-adherence to directly observed therapy instructions forced healthcare staff to allow clients to take SP at home. Need for investigating in improving adherence to IPTp administration was emphasised.

**Conclusion:**

High acceptability of the IPTp strategy at district level is meaningless unless necessary support is assured in terms of number, skills and motivation of caregivers and availability of essential supplies.

## Background

Globally, 216 million cases of clinical malaria and 655 thousands of deaths have been recorded in 2010 and a wide range of the literature shows that malaria is the major cause of morbidity and mortality in tropical and sub-tropical regions around the world. However, malaria is mostly concentrated in sub-Saharan Africa (SSA) where it accounts to over 85% of all cases and about 90% of all deaths recorded globally [1]. While all the people living in these regions are at risk of being infected, pregnant women and children under five years of age (especially infants) are the most vulnerable groups [2]. Various authorities have directed their efforts towards reducing the burden of malaria which has catastrophic effects on household socio-economic development and leads to a regrettable retardation of national economic growth. Only very recently research has brought good news regarding decline in number of malaria cases and deaths in several regions across SSA. This success is related to the efforts or initiatives made by government authorities in collaboration with private sector agencies and development partners to promote the methods which have already proven to be cost-effective for the control of malaria. In many countries, the most popular interventions implemented either separately or in combination include indoor residual spraying (IRS), promotion of effective use of insecticide-treated nets (ITNs) and intermittent preventive/presumptive treatment during pregnancy (IPTp), as well as environmental vector control measures. Emphasis has also been put on educating the public on the importance of early diagnosis and prompt treatment of suspected or confirmed cases using appropriate drugs. Meanwhile, intermittent preventive treatment in children (IPTc) is being considered/implemented in several countries particularly in areas with highly seasonal malaria transmission. This intervention is said to be a safe, efficacious and cost-effective if appropriately used [1,3]. A similar strategy known as intermittent preventive treatment in infancy (IPTi) was being investigated, but so far it has not been officially implemented as a national strategy in specific countries [1]. Considering the notable successes and anticipated benefits of newly proposed strategies, the national malaria control programme (NMCP) in Tanzania as in other countries has, with support from (or in collaboration with) development partners, shown a great determination for initiating or supporting all programmes aimed for leading to malaria elimination. The road to accomplish this elimination strategy is not easy, as challenges prevail, albeit with variations between one country and another and even within same country regions. Among the key challenges countries have been facing, is the scarcity of resources especially the money for financing the priority interventions/programmes sustainably and in the changing malaria epidemiology situation [1,4]. Other factors preventing the optimal delivery of basic health services including those aimed at preventing malaria are related to broader health systems issues such as poor governance, scarcity of human resources by numbers and skills, human resource motivation, the complexity of policy guidelines in terms of their formulation and interpretation, as well as the shortage of basic medical supplies and equipment [5,6]. Others include social-cultural factors influencing people to accept or refrain from attendance to health care facilities [7,8]. Yet, quite a few studies have been or are being published on the effectiveness of IPTp and of malaria prevention strategies during pregnancy in general. Therefore, more evidence is required for guiding the policy design and implementation strategy. In Tanzania, malaria in pregnancy (MiP) has been, and is still a major public health problem [4]. For many years, malaria control issues have been an integral element of district health service planning and management system [9]. In 2001, the government through its Ministry of Health (MoH) officially introduced the IPTp-SP policy as one of the key element of the focused antenatal care (fANC) strategy [10,11]. In the same year, the government announced SP to officially replace chloroquine as the national first-line drug for the treatment of uncomplicated (non-severe) malaria [12]. The targeting of IPTp-SP to pregnant women attending ANC clinics was in conformity to the Abuja Declaration of 2000 whereby the African Heads of States signed an agreement targeting to ensure that at least 60% of all pregnant women attending ANC clinics were able to access at least two doses of SP for IPTp against malaria during their pregnancy period by the year 2005 [13].

In its guidelines for IPTp, the World Health Organization (WHO) has recommended the first dose of SP for IPTp (popular as IPTp-1) to be taken by pregnant woman immediately after quickening (during the second trimester) and the next dose to be taken after one month during the third trimester. It was made clear that more than two doses of IPTp-SP can be administered per pregnant woman so long as there is a one month interval between one dose and another. This depends on the timing of the first dose. Thus, the WHO’s revised guidelines suggesting that more than two doses of IPTp-SP are allowed to be taken/administered under the directly observed therapy (DOT) system has helped to resolve the debate which has prevailed for several years regarding the benefit of the administration of more than two doses to the pregnant women who are HIV negative. However, the revised guidelines, prohibit SP to be given to HIV positive women using contrimoxazole and to administer folic acid/ferrous tablets concurrently with SP to pregnant women [14,15]. In conformity to the latter guidelines, the Tanzanian fANC guidelines require the first dose (IPTp-1) to be taken during the period lying between 20^th^ and 24^th^ week of pregnancy and the second dose (IPTp-2) between the 28^th^ and 32^nd^ week [10]. The IPTp policy requires the administration of IPTp-SP doses to be done by qualified healthcare workers (HCWs) under DOT [13]. This has not always been the case since reports from past studies and reviews in Tanzania show that there have been some practical challenges among which are the various socio-economic and systemic constraints [11,16]. As a result, the government’s goal of ensuring universal coverage of basic social services including health care services to all populations had remained more on policy papers than in practice on the ground. One of the strategies considered to be important toward achieving the latter goal has been the recommendation for all the health service providers to deliver basic maternal and child health (including ANC services) for free to all citizens visiting health facilities regardless of their ability to pay and other socioeconomic statuses. The evidence from systematic studies and reviews has shown that in some places health facilities (HFs) are non-existent while in places where such facilities can be physically reached, the services required by the clients are either missing or not always been delivered or accessed free of charge [17-20]. Reports continue revealing that the occasionally, the public HFs have been facing drug stock-outs and stock-outs of other essential supplies e.g. antenatal care cards. This has forced the frontline HCWs to instruct/advise the clients who fail to access these services to seek such services at alternative places such as contacting the private sources. This has been a disappointment to a considerable number of the patients and other community members [16,20].

The fANC strategy in Tanzania which was introduced in 2004 aims at minimizing the deficiencies shown by the previous/traditional ANC service delivery system [10].

In short, fANC differs from traditional ANC services in that the former has led to emphasis on the reduction of the recommended number of formal ANC visits during pregnancy from one month to where possible a total of four visits throughout a woman’s pregnancy. The fANC’s introduction was aimed at helping to reduce the workload to the existing few HCWs at HF levels and increasing pregnant women’s compliance with ANC services that would lead to safer pregnancy and delivery outcomes including better health of the mother and her foetus as well as the health of the baby [10,21]. In Tanzania, fANC was expected to improve ANC attendance rates. Already records show that in many places of Tanzania, ANC attendance is already as high as at least 80% for the women visiting clinic at least for one visit. Nevertheless, there are variations in the coverage IPTp doses taken by pregnant women attending ANC clinic in different country settings. These variations in IPTp coverage are related to frequent or occasional stock-outs of SP at HF levels, late or irregular ANC attendances by some women, and poor data recording and reporting systems at HF and district levels [22-25]. Other factors include inadequate stakeholder involvement in programme implementation [26], official or unofficial user-fees, weakness/lack of funds and commitment for health service supervision [11], and human resources shortages [16,26]. These reports have acted as a stepping stone or ground providing a good basis for investigating further on the feasibility of the IPTp-SP policy strategy’s implementation at district level. The investigation focuses on the operational effectiveness of the strategy (depending on its practicability and acceptability) from the perspectives of different stakeholders. There is a general lack of evidence on how different stakeholders especially those involved in the planning and implementation of the strategy do recognize IPTp-SP from their own experiences and perspectives. Among such stakeholders are the district health managers as well as other executive officers at district and national levels [5]. In attempt to narrow the current knowledge gap on this issue, the present paper presents and discusses the findings from a major study which was carried out to assess the economic and other contextual determinants of acceptability and practicability of IPTp-SP in Tanzania. The study used different methodological approaches, targeting different participants. Among those targeted were the district council health management team (CHMT) members, national level officers, frontline health care givers/HCWs, and target fANC service users (pregnant women). The main interest was in obtaining such stakeholders’ opinions regarding the status, and determinants, of operational effectiveness of the national IPTp-SP policy, and their suggestions/opinions on how the predetermined policy objectives such as those aimed at achieving high coverage of IPTp could made possible [27,28].

## Methods

### Study design

As highlighted in the foregoing section, this paper originates from a larger cross-sectional case study that was carried out in two districts, namely Mkuranga and Mufindi, in Tanzania. It was part of the PhD training programme of the first author of this paper [28]. From this training, a thesis has been produced and successfully defended in January 2010 at the University of Copenhagen [29]. The study districts are located in different regions which are far from each other, with different malaria transmission intensities and some different socio-economic characteristics and HF infrastructural profiles. More details on the characteristics of these districts have been described elsewhere [16,20]. The overall study mobilised IPTp-SP services data from multiple sources. These include the district CHMT officers/members [27], frontline HCWs [16], current and recent ANC clients [20] and national level officers working at the Ministry of Health and Social Welfare [28]. The study was exploratory in nature and adopted a case study design [30]. Since the viewpoints of other stakeholders mentioned have already been published elsewhere, the present paper concentrates on the qualitative data based on the experiences and viewpoints of district CHMT members in the two study districts. Although the data reported were collected between November 2005 and December 2007, the supporting or contradicting experiences and views expressed by other stakeholders are provided to enrich the discussion reflecting the diverse experiences/perspectives and to allow lessons to be learnt from similar or different experiences and insights.

### Study area and population characteristics

Mkuranga district is located in Pwani (Coast) Region in the southern part of, and nearby Dar es Salaam region. Mufindi district is located in Iringa Region in the southern highlands of Tanzania. According to the District Council Comprehensive Health Plans (CCHP) for Mkuranga which was reviewed during this study, the proportion of pregnant women who were registered at the ANC clinics as those who received IPTp-1 was 64.8% while those who received IPTp-2 was 42.3%. Records from the routine health service registers (popularly abbreviated in Kiswahili as MTUHA) indicated an average of 60.6% and 42.6% of the ANC clients were registered in Mkuranga to have received IPTp-2 in 2004 and 2005, respectively. Meanwhile, the Mufindi’s CCHP data indicated coverage of pregnant women amounting to an average of 58.2% and 56.7% for those registered to have received two doses of IPTp i.e. IPTp-2 in 2004 and 2005, respectively. In both districts, variations in the availability of monthly data were observed as shown by the HF records (particularly in MTUHA). As said above for the socioeconomic characteristics, additional details on these districts’ health, demographic profiles and socio-economic characteristics have been provided elsewhere [16,20].

### Study participants

The district CHMT members involved (in short, health managers) were included because of their being the ones who are responsible for conducting health service planning, supportive health service supervision, monitoring and evaluation, as well as performing other managerial duties at district level. Their experiences and opinions by virtue of their position as health managers were considered to be important since the district is the point of focus in the implementation of various health programmes within the national decentralization framework related to the health system and local resource allocation [11,31-33].

Within each target district, the CHMT members who participated in the study are as listed below. In total, about 8 district officers among those working as core CHMT members and those working as co-opted members to the CHMT were covered in each district (Table 1).

**Table 1:** CHMT members approached in the two study districts in Tanzania for a study on the feasibility of malaria IPTp with SP at district level

**Designation/Title of officer**	**Mkuranga district**	**Mufindi district**
•District Medical Officer (DMO)	√	√
•District Medical Officer in charge (i/c) of the District Hospital (DHMO i/c)	√	-
•District Nursing Officer (DNO)	√	√
•District tuberculosis (TB) and Leprosy Coordinator (DTLC)	√	-
•District Health Officer (DHO)	√	√
•District Health Secretary (DHS)	√	√
•District Pharmacist (DPharm.)	-	√
•District Laboratory Technician (DLT)	√	√
•District RCH Coordinator (DRCHCo)	√	√
•District Malaria and IMCI focal persons (DMICI-fp)	√	√

The cadre of District Malaria and integrated management of childhood illnesses (IMCI) focal person (Fp) and DRHCo are ones being co-opted (i.e. not-permanent members) to CHMT. The cadre of DMIMC-Fp was introduced not long time ago; According to Hildegalda Prosper, Janet Macha and Josephine Borghi, in their report to the Consortium on Equitable Health Systems (CREHS) of January 2009, the post of DMICI-Fp was introduced by the Ministry of Health and Social Welfare (MoHSW) in 2004 to assist the CHMT on issues related to data collection and reporting on malaria and IMCI in the country’s health care system. The aim was to strengthen malaria and IMCI activities in all districts in the country and the candidates recruited for this post were made to undergo training for 12 weeks in such areas as basic epidemiology and statistics; malaria and IMCI implementation; health information; management and research; managerial skills, planning, monitoring and evaluation; information, as well as education and communication/behaviour change communication. Some of the officers approached for study could not be reached (i.e. were missed) because of their being either officially occupied or on leave. Therefore, they were represented by the views given by other officers including the DMOs and DNOs, apart from the views expressed by several other participants.

### Data collection methods

The original plan for this study was to conduct focus group discussions (FGDs) with CHMT members in each district but practically this was possible in Mufindi only. In Mkuranga, the CHMT members preferred to participate individually through in-depth interviews (IDI) after finding it difficult for all of them to assemble for a FGD. Nevertheless, the themes covered under the FGD were the same as those addressed in the IDIs conducted in Mkuranga. What was actually done by the study team was to rephrase the themes covered in the FGD guide used in Mufindi and turning them into an IDI guide. This allowed the investigation to be performed on same issues for easier analysis and triangulated interpretation purpose. After noting that some of the officers approached were reluctant to give their opinions on particular issues in Mufindi during a FGD, particularly those which required one to be well informed of specific management issues, it was found reasonable to approach them separately later using the same IDI guide as one used in Mkuranga. This approach was supported by the officers concerned and these include DMO, DNO and DRCHCo, and DHS; and DPharm and assistant DPharm who expressed willingness to (and indeed did) provide additional managerial information in their areas of operation at office. The FGDs were recorded on tapes after receiving participants’ consent, but the recording system was corroborated with notes taken directly by hand during the discussion sessions in the study fields. The languages originally planned to be used was Kiwahili which is the first national language and for those who preferred it to English. However, the study participants were allowed to respond even in both Kiswahili and English (i.e. using a mixture of both languages) or use only English throughout for those who found it more convenient to do so. The use of some mixed words of Kiswahili and English was found to be more preferred and a common practice among health professionals interviewed elsewhere in Tanzania on similar issues in preparation for the main study mentioned above [11,27].

Study themes addressed in the FGDs and IDIs related to the determinants of ANC attendances, appropriateness and practicability of providing IPTp services including DOT, effectiveness of outreach or mobile ANC services, number and skills of the existing HCWs who were participating in the routine provision of ANC services and their motivation at workplaces, supply of SP for IPTp, and the effectiveness of PPP in the ANC service delivery system. Other issues addressed include the participants’ experience with public perceptions on the IPTp-SP policy and implementation strategy; the socio-cultural factors fostering or hindering pregnant women's effective utilisation of ANC services; and decentralization of financial planning and financial management at district level and its relationship with the planning and implementation of malaria control activities, including IPTp-SP. The operational words 'effective’ and 'effectiveness’ as used in this paper and in relation to the study objectives are taken to mean 'something e.g. a situation actually happening on the ground and in the manner as one would have expected or desired it to happen’ and this is consistent with various definitions which are found online (in the internet).

### Data processing and analysis

Tapes obtained from the FGDs were transcribed on the same day immediately after data collection in the fields. These, along with the handwritten notes taken during FGDs and IDIs were checked for their substance in terms of their relevance to the final report, guided by the study objectives. The notes were further qualified where necessary by replaying the tapes for listening them again while taking the notes verbatim and care was taken to consider the answers obtained from the follow-up questions asked to the informants for clarification. This was done so as a way of supplying the data collected with any missing pieces of information [34,35]. The notes taken in Kiswahili were later translated into English. Data analysis was conducted continuously allowing for information gaps to be addressed by returning to the interviewees as it deemed necessary. As recommended in case studies involving qualitative techniques, this involved making reference to the information collected from other study population groups [32; 38]. Data were analysed by looking at the content and pattern of the responses obtained by adopting a qualitative content analysis approach [30]. This involved going through the field notes and transcripts made out of the record taped FGD manually, systematically, and repeatedly until satisfactory output was extracted from the data. Key information was organized thematically in light of the study objective and the text extracted. Unquantifiable codes were attached to specific text units and sorting and comparing them in accordance with their relevance to specific study objectives. Similarities and differences of the situations described in the text units were noted and used to qualify the diversity of respondents' viewpoints.

### Ethical considerations

Ethical clearance for the study was obtained from the national Medical Research Coordinating Committee (MRCC) through the National Institute for Medical research (NIMR). Relevant regional secretariats and CHMTs were informed before the start of the study, and were provided with official letters from the central level authorities approving the study. Information was collected by a team involving a health economist with postgraduate education in other disciplines of management including business administration and health policy, planning and management. The team also comprised of sociologists and social workers, a professional educationist, and a pharmacist. The study participants were assured of their freedom to participate or not participating in the study voluntarily, confidentiality of the information they would give if it deemed necessary, right to withdraw from the study any time without facing any negative consequences, and were informed of the use of the study reports including various ways of dissemination. Majority disliked being record-taped during their participation in interviews and their suggestions were respected by the team which decided to take the field notes by hand only. No concerns were raised about tape-recording among the FGD volunteers. Participants provided written consent to participate in the study and agreed the study’s information dissemination strategy including its publications for wider public, research and policy uses.

## Results

### Factors associated with the quality and effectiveness of ANC and IPTp implementation

The most important factors reported to be associated with the quality and effectiveness of ANC and IPTp implementation as perceived by district health managers interviewed are identified (Table 2) and explained. As listed, these factors have been categorised in the analysis as being either systemic or service-provider related on one hand or user-related on the other hand. By user-related, consideration has been made of such issues as the perception of the user about drugs (including SP), timing of reporting at the ANC clinic by the pregnant women concerned, quality of services at HF level, and HF infrastructural environment compromising the quality of services. The service-provider related factors take into account of such issues as working environment, understandability and practicability of the policy guidelines, opportunities for at least short-term training on fANC issues, availability of funds for meeting essential supplies, to mention some. Drawing a line separating between service-related and user-related factors was not easy. At times, as it appeared, the service providers and service users could address the same issue and the only difference lied in the way they argued about the issue concerned.

**Table 2:** Factors associated with the quality and effectiveness of ANC and IPTp implementation in Tanzania as perceived by CHMT members in Mkuranga and Mufindi districts

**Factor**	**Systemic/service-related**	**User-related**
1. National funding for district health services	**√**	
2. Government support to ANC, IPTp and other health services	**√**	
3. Effects of drug policy change on user acceptability of SP	**√**	
4. Safety and efficacy of SP as perceived by health professionals	**√**	
5. Safety and efficacy of SP as perceived by pregnant women	**√**	**√**
6. Availability of SP for IPTp in public and private health facilities	**√**	**√**
7. User fees for IPTp at private and governmental health facilities	**√**	
8. Quality of IPTp services	**√**	**√**
9. Late registration and irregular ANC attendance of pregnant women		**√**
10. Quality and quantity of health facility staff and infrastructure	**√**	

The extent to which these factors are systemic and service-related or user-related (or both) is indicated with tick marks. Details are provided in the text.

### Perceptions about national funding for district health services

Respondents acknowledged the existence of the national decentralization policy allowing the district CHMTs to do their own planning of health service activities. Quarterly disbursements of funds for health planning were being provided from national level to district councils through what is called the Basket Funding System – a kind of sector-wide approach (SWAp) to financial planning [33,36]. Under this system, financial allocations are being approved by the Special Basket Funding Committee which includes representatives from the government and external donors. In order to receive funds under this system, district councils must present their annual health plans to the committee and have such plans approved. According to the DMO in Mufindi, private HFs could also receive financial support through the Basket Funding System, provided that budgets are presented, justified and approved. The funds allocated could be used to perform necessary procurements to cover some of the operational costs as per their institutional plans.

Late reception of funding from central level, and sometimes approval for their use at district council level, were viewed as a common cause of postponement of planned district health sector activities and a major challenge to the CHMTs who had to make difficult priorities of health activities on a short notice. ANC outreach activities, and thus IPTp services, is costly and logistically demanding, and therefore often sacrificed in this prioritisation process. The same applies to supervisory visits paid to the HFs. One of the implications of such a prioritisation is that HFs are visited less often than once per quarter, which is the targeted frequency according to national guidelines. Therefore, any practical challenges including service malpractice (e.g. in the administration of IPTp-SP) or shortages in human resources and other essential supplies existing in the health system regarding implementation of IPTp and other health services could not be mitigated in time or addressed completely.

### Perceptions about government support to ANC, IPTp and other health services

All respondents appreciated the IPTp strategy as an essential intervention toward reducing the burden of malaria in pregnancy, arguing that without this intervention the malaria morbidity and mortality situations at HF and community levels might be even worse than it is today. They acknowledged reception of the funds from the central government supporting district CHMTs to implement several planned health activities. Some of these funds have usually been spent for recruiting and training frontline HCWs involved in the delivery of IPTp doses and fANC in general. Also, acknowledgement was shown about the support received from the MoHSW to equip HFs dealing with fANC and other RCH service issues at district level. This related to the distribution of fANC guidelines, anti-malarial drugs (including SP), and other drugs e.g. antipretics for SP or other medicines, folic acid/ferrous tablets for anaemia control, etc., apart from some medical equipment, lab reagents and vehicles for mobile/outreach clinic services in peripheral areas. The vehicles and funds are also reported to enable frontline HCWs and district CHMT members attend seminars and workshops addressing RCH aspects including the issue of malaria in pregnancy (MiP). That way, the trained staff gain additional/new insights and skills. Nevertheless, the funds were reported to be inadequate, sometimes arriving late and therefore lading to postponement of some activities and affecting services.

### Perceptions about the effects of drug policy change on user acceptability of SP

The majority of the informants were concerned about the reactions of pregnant women towards the use of SP for IPTp. This concern was connected with the fact that the government has announced a replacement of SP with ALu (or coartem®) as the first-line drug for treating uncomplicated malaria. ALu was officially introduced for that purpose in the national health-care system in November 2006, but SP prevailed for IPTp purpose [37]. Yet, the participants from both districts reported that only limited reactions had been experienced. Suggestion was made that dissemination of proper information regarding the decision by the government of Tanzania to change the drug policy could have calmed the pregnant women and even the frontline HCWs and general public who seemed to doubt about the rationale for maintaining SP. The CHMTs took some measures to spread the news to the public using local government leaders, though challenges were still faced regarding the perceptions pointed out about SP. In Mufindi, the DMO commented: “*We work with local government leaders who are community representatives supposed to pass such messages to the people. We have informed the Ward Councillors through a special ward meeting concerning the national antimalarial policy drug change currently in the pipeline and the challenges we are facing with such a change*”. One of the challenges reported was related to people’s perceptions about the policy change. This seemed to contribute to the decreasing confidence in SP expressed by some ANC clients, particularly when it comes to taking IPTp-SP. In Mkuranga, specific arrangements to use community leaders to inform their community members about the drug policy change were not reported.

### Perceptions about the safety and efficacy of SP among health professionals

DMO and the majority of the CHMT members in Mufindi expressed confidence in the efficacy and safety of SP for IPTp despite their not being aware of any clinical trials that has ever been conducted in the district to confirm the efficacy and safety of SP. Their counterparts in Mkuranga were less confident in SP for IPTp. This is due to what they reported to be their experience with patients presenting malaria conditions shortly after taking SP. As one co-opted member of the CHMT and the DNO for Mkuranga reported, doubt about SP’s efficacy even for IPTp was based on the experience of the HCWs with the presence of a number of the ANC clinic attendees who after being found with malaria symptoms came to be confirmed later on through lab test as being malaria parasitemic within a short period after taking IPTp. In contrast, the DMO for Mkuranga viewed that SP was still efficacious and safe if timely and appropriately used, in spite of the evidence available that its efficacy against P.falciparum malaria was continuing to decrease in many places in the country. The reporters in the present study claimed to have been informed through the messages with evidence that were conveyed at malaria workshops and scientific conferences [38,39], and it did not take a long time that local newspapers began to report the same.

As illustrated by the following quotes, five CHMT members in Mkuranga had observed adverse effects in clients taking SP and expressed some concern about its safety:

*When they receive SP at ANC clinics they are advised to eat well and take adequate water*, *but those who do not have any of these materials end up feeling uneasy after taking SP and do think that it is the SP they have taken that is bad* (a CHMT member).

*Although it is recommended, SP seems to cause allergy to some people unlike chloroquine that never caused such seriously undesirable effects. To be frank, SP does not cure some of its users, so I doubt if the reported benefits are real* (a RCH staff, co-opted CHMT member).

Side-effects SP associated with adverse treatment outcome such as skin reactions, rashes, itching, vomiting and diarrhoea were occasionally reported, but in both districts, the Steven Johnson Syndrome (SJS) was reported to have been experienced in very few individuals.

### Perceptions about the safety and efficacy of SP among pregnant women

CHMT members in Mufindi had communicated with pregnant women who expressed concern about taking SP for IPTp. Nevertheless, majority of CHMT members were of general view or experience that SP was still highly accepted and the community still showed trust in it as an effective drug for malaria treatment. This high level of acceptability was perceived to be a result from the proper sensitisation of pregnant women about the positive effects (benefits), the limitations and the side-effects of IPTp-SP and use of SP for the case management of malaria. Sensitisation was provided regularly at the HF based clinics and during the ANC services delivered in the outreach community settings by the frontline HCWs who are assisted by village-based health volunteers. The common practice of self-medication with SP at community level was believed to stimulate overdosing and cause adverse reactions. As a result, it was argued, such practice could be a common cause of negative attitudes among community members or pregnant women towards SP in general. This would be particularly more problematic if SP were taken at home (e.g. for self-treatment of uncomplicated malaria) prior to or shortly after being offered SP for IPTp at an ANC clinic. It was thought to be difficult for the HCWs to get a feeling about the magnitude of this problem because of widespread public awareness that self-medication was disfavoured by the health authorities. CHMTs for both Mkuranga and Mufindi reported to have not been able to overcome this challenge in support of the efforts to scale up IPTp services and coverage rates proposed in Tanzania. However, the officers concerned in these districts acknowledged the NMCP for continuing to put emphasis on health education and community sensitization throughout the country. In contrast, the concern about SP’s preventive treatment for IPTp and curative potential for case management of malaria seemed to be high among the frontline HCWs and pregnant women interviewed within the study HF settings and among lactating mothers and pregnant women who were confronted during FGDs held at community level.

### Availability of SP for IPTp and existence of user fees in public and private ANC clinics

Essential drug kits are distributed to governmental HF by the DMO’s Office on regular quarterly (and sometimes monthly) basis, and for this reason, SP is rarely out of stock in governmental (public) HFs. Non-governmental (faith-based or commercial private HFs), however, were not receiving the essential drug kits (and thus SP) from CHMTs with the same degree of regularity as government/public HFs were experiencing. Therefore, that is why the stock-outs of SP at such HFs were occasionally occurred, thus hampering delivery of IPTp-SP for free to the clients as recommended. Majority of the interviewees and FGD participants in both districts viewed this tendency of the service providers to charge their clients for drugs as contradicting the government policy of promoting universal access to basic healthcare services for free, be it at private HFs or at public HFs. Based on requests from private HFs suffering from SP shortage, excess supplies of SP at governmental HFs have been often transferred to private facilities at no cost. When such requests are not met, ANC service providers were found being forced to ask the pregnant women to procure SP for IPTp elsewhere.

Appreciating the role of private service providers, one of the staff found working in the RCH aspects at district level and who was a public health nurse in Mkuranga commented: “*Some of us try to advise the district pharmacist and other responsible officers to reconsider supplying private HFs with SP for IPTp. For instance, Kiparang’anda-Arafa dispensary is very important to us since it is trusted and attended by many pregnant women. Sometimes, it attracts more women than those we receive here at the district [government] hospital*”. This point was validated by the reports from the HCWs who were found at the said dispensary. The research team confirmed this situation through direct observations on the high number of the clients attending at that dispensary and several other dispensaries (private and public) on some working days in both study districts. A few clients through individual interviews claimed that they wanted to save the time of staying at the clinic waiting for the service as they were used to face troubles when seeking service at the district hospital. They also reported to enjoy the courtesy of the staff working at the latter dispensary and that they could hardly enjoy the same if have visited the district hospital.

Furthermore, CHMT members in both districts confirmed that pregnant women were occasionally paying for SP given for IPTp purposes at the private HFs especially when there were no subventions from the government side. It was added that even when SP was readily available at these facilities, respondents in Mkuranga confirmed that pregnant women were often being asked to pay money for the IPTp with SP. In Mufindi, the majority of the respondents were less certain about whether or not user-fees for IPTp existed in practice even both at government and private HFs. However, the CHMT members from both districts confirmed to have been receiving concerns/complaints from community members or their leaders about the reported/perceived official and non-official user fees imposed on pregnant women for ANC services delivered at government HFs. They insisted that coming up with proof about the reported unofficial fees e.g. payment under the table was very difficult for the CHMT members who were not routinely working directly on health service delivery. Implementation of such fees at private HFs was viewed to contribute to poor IPTp adherence. One of the public health nurses in Mkuranga commented: “*Occasionally during my supervisory visits, I have observed the ANC cards of the women who have not been given SP and other services. Feeling sympathetic to such women, I have been intervening to ask the health personnel to do the best they could to offer necessary services*”. HCWs also testified in relation to the actual existence of user-fees for ANC services. This includes paying for SP administered or prescribed for IPTp as reported by pregnant women and lactating mothers in both districts. However, the research team came to note later on that some of the so-perceived ANC fees were not specifically directed to ANC services only or at all, but were imposed to every client contacting the clinics concerned as a way of covering at least part of the operational charges (OC) which the facility management incurs. For instance, participants could pay a little registration fee for procuring patient card, paying watchman’s wage, paying for HF utilities (water, electricity, charchoal for various boiling activities done at HF level in favour of patient services, or lunch for outreach/mobile clinic staff, etc.). These payments were passed by the HF management committee formed by the community representatives among which were village leaders and HF committee staff.

Although the commercialization of health services was one of the main reasons for suspending the distribution of free SP to private HFs, one DRCHCo viewed that failure of the district pharmaceutical unit to supply the private HFs with SP was unjustifiable. This is because doing so might deny the pregnant women their right to access free SP for IPTp as recommended by the government through its IPTp policy. Denial of women’s right to access free IPTp-SP would impose unnecessary risk to such women of contracting MiP and therefore lower the national prospects for attaining the goal of covering at least majority of pregnant women with IPTp. The examples given about the effective collaborative efforts between governmental and private health service providers include the national immunization/vaccination and TB treatment programs, which have been helpful in the implementation of government health policies since independence. However, a respondent in Mkuranga challenged the private HF personnel or management for often failing to submit their reports in time to the CHMT where they could indicate drug shortages faced or requirement needed over time. In addition, DMO observed that the quality of CHMT supervision to peripheral public and private HFs had serious deficiencies both in terms of data inconsistencies, incompleteness and lack of key details that could be useful when it comes to effective monitoring and evaluation and eventually guiding district health service planning and reporting to central level in Dar es Salaam. As such, a higher degree of responsibility in reporting should have captured the need for more supplies, including SP. It would also help the DMO’s Office to consider alternative ways for fostering collaboration and liaising with other authorities such as the MSD (Medical Stores Department and MoHSW) to ensure sufficient stock of the required supplies all the time. At HF level, HCWs in the frontline expressed concerns that the supervision performed by CHMT members is occasionally done in haste in such a way that the supervisors fail to recognise all the pressing needs of the HF staff [33]. This point was not challenged by CHMT members who argued that frankly speaking the supervisors from the district level cannot avoid this to happen as they are forced to do so. They usually find themselves under pressure of a tight working schedule. The challenge becomes more intense when a CHMT member has to visit some facilities in a particular quarter but due to financial or vehicle shortages as well as other official events interrupting their schedules the activities are not implemented as desired.

### Perceptions about the quality of IPTp services

Respondents in both study districts generally agreed that adherence to DOT for IPTp-SP administration had improved over the past four years. This was considered to be a combined result of increased quality control of ANC services, coherent advice provided during CHMT supervisory visits, and in-service training related to fANC. It was felt that the inappropriate practice of allowing pregnant women to take SP at home instead of under observation at ANC clinics was effectively declining. Some respondents argued, however, that the DOT approach socially forced doubting pregnant women to accept swallowing SP tablets against their will. The view on DOT practicality was supported by reports from current and recent ANC clients as well HCWs in both districts. Testimony was given about some of the clients who delayed to attend clinic as a way of escaping from timely reception of SP for IPTp. CHMT thought that this was a problem requiring a multi-sector approach to address rather than relying more on the simple health education methods implemented at HF level involving frontline HCWs and on occasional periods their supervisors from the district level. In contrast the view that IPTp administration has improved due to staff being more trained on fANC aspects was opposed by majority of the private sector HCWs.

The report from the frontline HCWs in Mufindi concerning the District Council supplying all HFs with bottled water processed at factory level in support of DOT system for IPTp-SP [16] was confirmed by the CHMT. This had diminished the need for clean cups that would otherwise be required by the client needing to swallow the SP tablets under DOT by a HCW. Funding for the provision of bottled water came from tax revenues collected from private tea and timber plantations and factory companies. In Mkuranga, the HFs frequently experienced shortages of clean drinking water, especially during the dry seasons. Unfortunately, the District Council in Mkuranga did not have the necessary financial resources to support HFs with bottled water, hence an inequality in the sources of income between the two districts which has a bearing on the feasibility of implementing particular interventions including IPTp. In effect, the respondents confirmed that HCWs in Mkuranga occasionally asked the pregnant women to bring along the drinking water when coming for ANC services. Alternatively, allowing those coming without water to take SP at home unsupervised at their own time seemed unacceptable and against DOT guideline. Some CHMT members agreed with the point raised by the interviewer about the possibility of including the bottled water item for IPTp in the Council’s Health Budget using either the health basket funds, or block grant from the central government level or revenue collected from user fees. This point was criticised by other members who doubted that the budget allocated at central level was too small to cover the item of water without complicating the matter further. This means that doing so might reduce the budget for other priority activities unless the total budget allocated from central level was increased.

The DMO’s of both districts did not underrate the fact that effective planning for health services at HF level involving the frontline HCWs at HF level and the community representatives might help to raise the issue of water shortage problem and possibly discussions on how it could be overcome with community and district council’s involvement. Meanwhile, two respondents including one from each district found it inappropriate to request the ANC clients carry their own water for taking SP under DOT on ground that allowing the clients to do so might be regarded add inconveniences to those living far in remote villages and therefore disappoint some of the clients to attend clinic. Yet, these respondents recognized and acknowledged that the practice of letting the clients bring their own water at HF level was common in the TB programme, as similarly reported by national level officers [40]. The shortage of cups for taking SP was not considered to hamper the practice of DOT in Mkuranga somehow. The reason given in justification of this situation was that not all of the clients (and especially the relatively highly educated ones) would feel comfortable to use the same cup by sharing with others for taking SP under DOT. Two respondents expressed concern about the health risks of sharing water cups if such cups were not properly cleaned. Indeed the HCWs reported the same to have been experienced especially among the school teachers and formal employees.

Incidences were reported about pregnant women who were hungry and thirsty after travelling for hours before reaching the intended clinics. Yet, such clients have to wait longer to be attended to at the clinic and being forced to swallow SP under DOT. The DMO of Mkuranga felt that it is not only a question or matter of supplying ANC clinics with drinking water, but also of ensuring that HCWs and their supervisors (e.g. CHMT members) are committed or motivated to perform their duties well and interact well with the clients courteously. The issue of giving SP to hungry clients was considered to be a systemic challenge beyond the ability of the DMO’s Office or District Council to address. Nobody seemed to disbelieve that SP could still be administered to clients with empty stomachs so long as the clients drink it with much water, and this is because the revised WHO guidelines for IPTp implementation allowing administration of SP to pregnant women with empty stomachs had not yet come out and instead they came out in 2012 [1].

### Perceptions about late registration and irregular ANC attendance of pregnant women

In both study districts late registration (i.e. within or after pregnancy week 20) and irregular attendance behaviour of pregnant women for ANC services was viewed as a challenge in the efforts to increase coverage of IPTp coverage. Apart from increasing the risks of complications during pregnancy and delivery, late registration and irregular attendance also contributed to undermining the reputation of ANC services. This is because the provider-client relationship becomes tenser as a result of pregnant women's poor adherence to services and recommendations. It was interesting to find the members admitting that late registration originated from a combination of factors -socio-cultural, economic, and health systems, and service related. CHMTs were also aware that registering late for ANC services one way of hiding an unplanned pregnancy among the teenage girls and even some adult women including those in marriage bondages. Among the factors known to hamper IPTp coverage as indicated by the responses include long travel distances to ANC clinics, bad weather such as heavy rains, a feeling of insecurity during travelling, and financial and time costs associated with seeking ANC services. These challenges were perceived to continue facing the districts and therefore affecting the strategies established with the aim of scaling up and optimising the delivery and uptake of IPTp doses.

Efforts tried out for overcoming the prevailing constraints as reported by CHMT members include educating pregnant women to visit clinics early and encourage them to be escorted by their spouses to the clinic so that the spouses could also get education on safe pregnancy and motherhood measures. Other measures include involving village health workers (community health volunteers) to move around and pass the message in the households. It was viewed in both districts that these efforts needed to be supported through multidisciplinary and multisectoral approaches at district level with support from the central level.

### Availability, skills and utilisation of HCWs at HF level and infrastructure

Acute shortage of staff, especially at peripheral HFs seemed to be the main obstacle to the effective IPTp service delivery in both study districts. Commentators acknowledged that staff working at the RCH clinics are often overworked and this lowered their ability to provide quality services throughout their official time. This way, the dissatisfied clients continued complaining against receiving what they perceived to be the services of poor/low quality. This fact was noted through such means as personal communication with community leaders or patients/clients at HF levels during health service supervision. In some HFs especially the smaller and more peripheral ones, the work pressure was intensified by very diverse set of professional and administrative duties of the few HCWs available, a point that was also stressed by the frontline HCWs interviewed.

In both districts, reports from CHMTs, which resembled those obtained from the frontline HCWs [16] showed that many peripheral HFs were suffering from limited office space. This includes the place for comfortably providing IPTp and other ANC services. As a result, inconvenience was being faced by the frontline HCWs during health education sessions held at once with many clients who attended clinic on particular days. Occasionally, the present study team observed some of the clients standing or sitting outside of the HF buildings while such clients were waiting to be attended by the HCWs. They seemed to be sad or disappointed when there was either high intense sunrays or when it was raining. It was not uncommon to find the ANC services being delivered in the same building/room where other RCH services were being provided. Thus cries of children and patients overcrowding at the HF eventually demoralised both the staff and clients.

Suggestions about establishing new buildings or expanding existing ones in an effort to harmonise the capacity of HFs with the number of clients and patients served as given by some members were challenged by other participants among the CHMTs in both districts. For example, the DMO of Mufindi viewed that larger HFs like hospitals and health centres would require more staff and resources for services and maintenance, and this was not realistic given the budget constraints in the health sector. The issue of inadequately trained staff number exceeding the number of those adequately trained on fANC and other service aspects was not denied by the CHMT officers who claimed that the measures being taken so far to improve the situation were not much effective to make a significant difference due to budget/resource shortages.

### Outreach ANC services and peripheral health supervision

ANC services are also provided as outreach services in remote communities where access to HFs is difficult are often provided along with child vaccination services at community level. As discussed above on the issue of health service supervision, reaching the optimum level of these services depends on the availability of adequate HCWs, transport and other basic supplies. Other limitations are related to lack of special buildings and poor communication network especially during rainy seasons. Using DMO’s official car or district hospital’s car or particular vertical programme’s car to support mobile clinic and outreach services was common due to vehicle shortages at CHMT level. In Mufindi CHMT members acknowledged what they called an improved road system that allowed the remote villages to be relatively more and easier to be reached throughout the year unlike in the past. In Mkuranga, CHMT members expressed their disappointment with the HFs located in remote villages particularly those surrounded by bushes in which dangerous wild animals including lions and hyenas dwelled and occasionally attacking or threatening people and frontline HCWs. These views were confirmed by the reports from frontline HCWs, pregnant women, lactating mothers and the observation made by the present study team of bushes surrounding such areas as Kisiju and Mkamba HCs as well as Magawa and Sotele government dispensaries.

Lack of vehicles or fuel or money for vehicle maintenance has been hampering the carrying out of mobile/outreach clinic services and general health supervision at HF and community levels. Yet the basket funds and block grant from the central level could not support this satisfactorily. Other respondents acknowledged there was a differential (discriminatory) treatment and support provided to government and private health facilities when it comes to budget allocation for other charges out of which the funds for supporting outreach services could be obtained. Use of private bicycles or other means of transport seemed to be a common cause of delay in the service delivery and compromising the quality of services at outreach clinic levels. This happened so because the HCWs had to rush their services in attempt to save their clients from waiting at the clinic for a long time on the days when the clinic was overcrowded by clients or when the HCWs arrived at the clinic late. One officer in Mkuranga had the following to say: “*At times health workers are injured from falling on their bicycles on their way to the outreach villages and some of the staff decide to go back without reaching their destinations, hence making their clients wait in vain and getting extremely disappointed, demotivated to visit the clinic again or ending up complaining to higher authorities*”.

### Suggestions for optimising the quality and effectiveness of ANC and IPTp services

(a) General Views

With regard to the measures suggested for the central and district level authorities to consider if they were to improve and optimise the delivery of quality ANC services and effectiveness of the IPTp intervention, the views were also obtained. In general, it was emphasised that community sensitisation on the risks associated with pregnant women’s late registration and irregular attendance to ANC needs to be prioritised and this should be an intersectoral collaborative task. The reproductive and child health (RCH) education which is increasingly being recognized by the government as an essential package of the curricula of primary and secondary school education as well as part of higher education require more involvement and commitment of various ministries. The key sectors include the ministry of education and vocational studies, ministry of finance and economics, MoHSW, ministry of community development, gender and children, and other actors in the public and private sectors including non-governmental organizations (NGOs). Moreover, intensified sensitization of pregnant women at community and HF level, possibly involving peers or other local community representatives, on the usefulness and safety of IPT-SP was also emphasized to be another useful way of building or enhancing confidence among the pregnant women in IPTp services. Local government leaders were considered to be among the important local stakeholders if involved in the campaigns for RCH services in the community, including the issue of IPTp. Meanwhile, committing funds for procurement of bottled water requires the central and local government councils both at regional and national levels to prioritize in order to support staff training programmes and HFs with the necessary supplies.

(b) More specific views

These are individual and more targeting suggestions emanating from or linked to the above broadly mentioned suggestions and they include those viewed to be easily implementable at different levels as outlined below:

(i)  *Central and district council authorities*

– The Central and Local Government Authorities in consultation with district CHMTs should ensure that health facilities delivering primary health care services are adequately equipped with skilled health service staff;

– The Ministry of Health and Social Welfare (MOHSW) should intensify its support to the districts by increasing the frequency of supervisory visits and the provision of SP, other essential drugs and other forms of technical and moral support to both governmental and non-governmental health facilities.

– The National Malaria Control Programme (NMCP) should intensify its monitoring of malaria services provided at district level including material and medical supplies, and the quality of services.

– The MOHSW should intensify its formal sensitisation and training of health workers on ANC issues. This is of particular importance when there is a change in national policies and implementation guidelines related to ANC and IPTp. The training should be carried out as an iterative process designed to cater for changing codes of conduct and staff turn-over.

– The MOHSW should work with other ministries and government authorities to strengthen safe motherhood training in primary and secondary school as well as in higher education.

– As part of their long-term development plans, the MOHSW should prioritise the construction of permanent water-wells at health facilities suffering from inadequate drinking water.

(ii)  *District CHMTs*

– CHMTs should allocate funds in the annual health budgets for procuring bottled water.

– CHMTs should prioritise the implementation of supervisory visits to both easily reachable and remote health facilities. These visits should provide moral, material and technical support. Challenges encountered during such visits should be reported to the district councils with suggestions on how to tackle them.

– Health officers involved in supervisory visits should be properly trained in how to supervise health workers, monitoring and evaluating the performance of health workers, providing technical advice, and capturing, analysing and tackling problems at health facility level.

(iii)   *Health Facilities and ANC clinics*

– Health facilities should take measures to reserve safe drinking water at health facility level for pregnant women who arrive at ANC clinics without drinking water.

– Health facilities with major water shortages should prioritise sensitising pregnant women on the need to bring along drinking water from home so that SP can be administered under observation in ANC clinics;

– Health facilities must intensify community sensitisation efforts regarding the importance of ANC services for safe motherhood including early registration, regular attendance, IPTp services and SP safety. Sensitisation efforts could involve local community members, peers, opinion-makers, role-models etc.

## Discussion

### Perceptions and attitudes towards the national IPTp-SP policy

This study makes a good contribution to the national and international literature by presenting important evidence regarding the perceptions of district health managers in Tanzania regarding the feasibility of IPTp services based on SP and provided through ANC services. Positively, the CHMT members in both study districts appreciated the national and international IPTp-SP policy as one of novel and essential pregnancy and newborn life-saving interventions. But based on their experiences, such officers anticipated that the public loss of confidence in SP for IPTp after SP’s replacement with ALu for treating uncomplicated malaria could undermine IPTp coverage. Indeed, a pervasive public loss of confidence in SP would be a major challenge to IPTp and thus ANC services if SP remains the drug of choice for IPTp for the unknown length of the future. So far there was, and still there is no drug alternative to SP for IPTp [14,41]. The relevance of this reported loss of confidence in SP as expressed by the district managers concerned in the present study as well as current and previous ANC users [20] is justified with sufficient scientific evidence. Research scientists have revealed that SP’s almost ten years of widespread use in many countries of SSA, uncertainties about the optimal dose remain [42]. Moreover, currently doubts prevail among the clinicians and other epidemiologists who have decided to undertake clinical trials for testing safety and efficacy of new drugs alternative to SP for IPTp e.g. mefloquine [43]. Meanwhile clinical trial assessing the cost-effectiveness and efficacy of SP when used in combination with and screening and treating the pregnant women at each ANC visit and encouraging them to sleep under ITNs could offer more chance for SP to continue being used for IPTp [44]. Also, the doubts showed by CHMT members about the ANC clients’ sustained trust in, and acceptance of, SP as an unsafe and less efficious drug are consistent with the evidence reported from other studies within and outside of Tanzania [12,45,46]. As viewed by some CHMT members, the possibility that messages attached to ALu’s promotion against SP as the first-line might have undermined public confidence in and attitude towards IPTp-SP. All these facts together with other evidence about the widespread parasite resistance to SP especially in Eastern Africa [38] imply that public debate about the sustained use of IPTp-SP will prevail and may even become more intensive. Even if an alternative/new drug for IPTp comes to be discovered following the ongoing clinical trials, there is need for continuing to promote IPTp with clear and convincing messages to direct service givers and users as well as managers.

### Concept of private public partnership in theory and practice

An interesting observation from the present study is that the concept of PPP in health service provision was appreciated by CHMT members in both districts. In particular their acknowledgement/perception of the need for the central government to support to private healthcare sector/providers when it comes to involving this provider in the policy requiring them to deliver free services to priority vulnerable groups. Yet, as shown by the reports from the present study group and other stakeholders [16,20], the private HFs were being rarely supplied with SP for IPTp delivery for free to the clients, hence indicating a systemic weakness in its own place. Under this situation, it may not be realistic to challenge the private care providers for imposing fees upon the SP given to their clients or referring such clients to seek SP elsewhere. Notably, non-governmental HFs were found contributing to as much as 35.7% and 26.3% of all HFs in Mkuranga and Mufindi, respectively, and around 40% of all HF in Tanzania [47]. This may result to be a serious challenge against the efforts made by the districts concerned and the government/nation to expand the provision of ANC and IPTp services in equitable manner.

With the high proportion of non-governmental/private HFs present in Tanzania, there seems to be a strong argument for integrating private HFs more deeply into the health care system that provides essential health services to the public. While the advocacy for having a stronger PPP in the health service planning and provision sounds very relevant, it is important to recognize that the process necessary for achieving the objective requires proper legal frameworks (laws or regulations and mechanisms for their enforcement), other specific strategies, and political will/commitment, and continuous sensitization of HCWs.

### Stock-outs of SP for IPTp at HFs and implications on IPTp coverage

It is clearly confirmed by the district health managers in the present study, as similarly reported by the national level officers [5], the frontline HCWs [16] as well as the ANC users [20] that even the public/governmental HFs have been facing stock-outs o SP for IPTp from time to time. Similarly, the shortages of SP in governmental HFs have been reported under the Tanzania National Voucher Scheme, Household and HF Survey Report of 2007. In that report, it was revealed that only 59% of the HFs had SP in stock on the day of the survey (Dr. Mufungo Marero, NMCP Officer, per comm). These shortages could be minimized if not at all avoided by allocating additional budgets from the basket funds to districts and allow HF authorities have autonomy to plan for their use and actually access such resources. Alternatively, CHMTs and HF authorities could be allowed to utilize the revenue collected from other sources such as cost-sharing. However, for this to be accomplished, still there must be more open and well guided criteria for achieving what has been planned.

### Late reception of funds from central level and implications on IPTp-SP delivery

The late reception of funds for district health services from central level appeared to contribute undermining efforts to optimise healthcare services in the districts. This also seems to have been a chronic weakness contradicting the predetermined district health budget targets or goals not only in Tanzania as reported before [31-33,48], but also in other places within SSA [49,50]. Thus, in event of such financial shortages partly influenced by national level actions, it is impossible to realise efficiency gained in the activities performed at district level, including those targeting for attaining a high coverage of pregnant women with IPTp-SP.

### Human resource constraints and requirements in relation to IPTp coverage/services

Tanzania continues to face chronic shortages of financial and health service personnel. Therefore strengthening the capacity for existing HFs including equipping HFs with adequate materials and skills and motivated health personnel might strengthen the health service provision system in general, and in relation to IPTp-SP [16]. The HCWs need support related to at least in-service short-term training including training addressing RCH and specifically fANC aspects [16]. Private sector providers should not be neglected or given more limited opportunities than the public sector providers. This is because doing so not only disappoints the private sector agencies to provide the services required, but also the community who contact such providers come to lose when they face the consequences such as inconvenience of missing some of the basic services they desired/needed. The training should cover as many personnel as resources conditions may allow. Otherwise, experience from other countries shows that the complaints about shortage of trained staff to either deliver or supervise administration of IPTp-SP by proper interpreting the national guidelines may continue if the few trained staff get transfer to work in other places [51].

### Uncertainties in real coverage rate of IPTp-SP beneficiaries and effects on service plans

CHMT members’ view that the data collected from HFs is not much reliable for confident planning and reporting is well argued and supported. A survey carried out in 21 Tanzanian districts observed that the average uptake of the IPTp-1 increased from 26% in 2001 to 65% in 2005, whereas the average uptake of IPTp-2 was 46% in 2001 and 45% in 2005 [52]. Marchant *et al*., [24] found that the proportion of pregnant women attending ANC clinic who received the IPTp-1 declined from 71% in 2005 to 65% in 2007, whereas that of IPTp-2 declined from 38% in 2005 to 30% in 2007. The latter authors noted that rural districts were significantly more negatively affected than urban ones. They also found that systemic factors including the availability of SP contributed to the observed differences. SP availability thus decreased from 85% in 2005 to 60% in 2007. These findings reveal the inadequately explained variations and fluctuations in the coverage rates of women receiving IPTp-SP in different country settings, therefore, suggesting the need for more evaluations to be performed. Across SSA the issue of actual coverage of IPTp is still being debated and subject to further systematic research [8].

### Effectiveness of DOT algorithm for IPTp-SP administration and compliance challenges

The failure in the practicability of DOT for IPTp-SP as reported by the CHMTs in present study as also documented from the views taken from the frontline HCWs regarding the dilemma often being faced in the clinics with no clean drinking water supply calls for deliberate attention [16]. The reported difficult choice between offering pregnant women, who do not bring along their own drinking water, to take SP at home or to refuse giving them SP since doing so might seem contradicting the DOT standard guideline, was well noted. However, both of these reactions are suboptimal and associated with health risks for the pregnant women and their offspring. The most common, ethical and safe reaction is to allow the pregnant women to take SP at home as this increases the chance that the mother concerned would swallow the drug and comply with the treatment regimen. Other more long-lasting measures need to be taken if the ambition is to improve the quality, coverage and effectiveness of IPTp services in Tanzania. On the other hand, effective practice of DOT seems to be undermined or hampered by such other factors as shortage of SP for delivery at health facility level and misinterpretation of the IPTp guidelines by the untrained personnel working in the ANC facilities. This is a challenge in other countries as well [51]. Without effective practice of DOT, there is no ground to justify beyond reasonable doubt or without certainty any statistics given about the IPTp coverage. For example, those allowed to take the drug outside the HF may not comply at all.

### CHMTs’ suggestions regarding the way-forward toward enhancing IPTp coverage

There are many good points among the suggestions, ideas and solutions presented out of this study. These include the issue of decision-makers at district level to consider making provisions for procuring bottled water for pregnant women taking IPTp-SP, intensifying community sensitisation on the nature and importance of IPTp and ANC services in general, improving water supply systems at HF level, as well as the timely, effective and adequate support to CHMTs by central (national) level authorities. These are relevant suggestions since they are consistent with those documented before [5,48]. Nevertheless, the solutions should be aligned with the exact nature and magnitude of the problem and with the realities on the ground. In one place the main priority may be to address the problem of water shortage, in another it may relate to geographical accessibility, inadequate logistical or technical support, or HF understaffing.

On one hand, we argue in support of views presented before by other authors that the Governments need to adjust the globally set targets for service coverage and health outcomes so that they can match with the realities and priorities at country level [53]. The IPTp policy’s practicability as many other health interventions depends on primary healthcare system which is already constrained by inadequate human resources, low healthcare staff motivation, health infrastructure, equipment and finances. This is a challenge since the success of any health programme depends not only on the efficacy of the interventions, but on the uptake of those interventions by the target populations [54]. In addition, the interventions concerned are implemented in the HF contexts whereby other specific and broader health system issues/challenges have been addressed [7,8,51]. Experts have warned against too high expectations on the health impacts of individual health interventions, for instance, IPTp in this case, in situations whereby such interventions are not integrated in the system along with other interventions existing in the system [15,55-57]. As ANC services are often delivered along with other health promotion and prevention programmes such as vaccination programs and child clinics [58], a careful consideration of the low compliance with and coverage of IPTp service provision to pregnant women in many African countries so as to find means for improving ANC services would be one way of enhancing IPTp compliance and coverage.

### Strengths and limitations of the present study

In order to be able to improve the situation, it may be better to learn from contradictory opinions/views about something. The perceptions expressed by CHMT members in this study reveal that such officers were not always in agreement with everything perceived by the HF based staff within the same two districts regarding the factors hampering or promoting the practicability of particular malaria interventions including IPTp. For instance, while the CHMTs acknowledged the support from the central government in relation to training of HCWs on fANC aspects, they did not point out the weaknesses expressed by the HWs regarding the criteria and processes used to select candidates for the training courses and seminars. In contrast we have seen that the HCWs on their part criticised the CHMT members and other organisers of the training programmes for being too much discriminatory in the selection of the course candidates from public and private sectors, thus disappointing the lower staff cadres especially those in the private sector [16]. Also, the CHMT members did not comment anything on the issue presented by national level officers and frontline HCWs that district CHMTs sometimes believed that private sector providers would sell the drugs if supplied them to deliver such drugs for free [5,16].

Furthermore, the main limitation to this study lies in the participants’ views on the issue of IPTp-SP low uptake. Such participants might have been influenced by their own feelings or speculations about SP as opposed to the real feelings of all pregnant women and the general public. One of the reasons in support of this comment is that the study was carried out shortly after the government had replaced SP with ALu as the first-line antimalarial therapy. Therefore, the time interval/period might have been short for CHMT officers to assess the health seeking and service delivery behaviours in their districts. However, the views of such officers were supported by several reports published in previous papers regarding community and service providers’ perceptions about SP and ALu in Tanzania [11,45,46]. Another key limitation of the present study lies in the use of different techniques to collect data from the same category or cadres of the CHMT officers from the two study districts. That is, the participants fall under the same category of designations or routine duties were let to contribute their opinions using different data collection techniques. It is appreciable that care was taken to ensure that the IDIs and FGDs addressed the same issues, but the fact that the mode of application of such techniques differed is subject to debate. The IDI participants could not get to hear from what others were saying on the same topic/issue while in the FGD case that was maximized. Meanwhile, the IDIs gave more freedom of the respondents to express themselves than those who participated in the FGD whereby both the seniors and their subordinates were in the same group. Thus, this approach possibly contributed to the opinions observed/reported to differ among the study participants. In light of these limitations, it is admitted that the findings from the present study, however useful they seem to be, should be interpreted cautiously.

## Conclusion

High acceptability of the IPTp intervention at district level is meaningless unless necessary support is assured to healthcare providers in relation to number, skills and motivation of caregivers and availability of essential supplies. Despite the district health managers appreciating IPTp-SP to be an essential and not merely an important strategy, they have a general feeling that additional support is required to allow a strong PPP in fANC services as in other areas of public health, timely disbursement of funds from central and council level and need for addressing broad systemic constraints. These include constraints relating to staffing levels and skills, supply of SP and other essential medicines, health service supervision, health information sharing for public sensitisation on fANC aspects and clear policy guidelines. It is true that if properly planned and implemented, the IPTp strategy remains to be an essential intervention against MiP in Tanzania.

## Competing interests

All authors have read and approved the paper for publication and declare no conflict of interest in relation to this paper.

## Authors’ contributions

As part of his PhD training, GMM conceived the study, participated in all stages of the study’s implementation, data management and analysis and study reporting which includes writing the first draft and final versions of this manuscript (MS). PB, PM and JB supervised GMM’s PhD study and commented on the first draft of this MS. PB was one of the two GMM’s lead PhD supervisors and provided more extensive comments on the MS. All authors read and approved the final MS.

## Pre-publication history

The pre-publication history for this paper can be accessed here:

http://www.biomedcentral.com/1472-6963/13/372/prepub
